# Virtual Reality Social Prediction Improvement and Rehabilitation Intensive Training (VR-SPIRIT) for paediatric patients with congenital cerebellar diseases: study protocol of a randomised controlled trial

**DOI:** 10.1186/s13063-019-4001-4

**Published:** 2020-01-14

**Authors:** Niccolò Butti, Emilia Biffi, Chiara Genova, Romina Romaniello, Davide Felice Redaelli, Gianluigi Reni, Renato Borgatti, Cosimo Urgesi

**Affiliations:** 1 Scientific Institute, IRCCS E. Medea, Bosisio Parini, Lecco, Italy; 2 Politecnico di Milano, Milan, Italy; 3 IRCCS C. Mondino National Neurological Institute, Pavia, Italy; 4 Laboratory of Cognitive Neuroscience, Department of Languages and Literatures, Communication, Education and Society, University of Udine, Udine, Italy

**Keywords:** Virtual reality, Social cognition training, Cerebellum, Paediatric patients, Rehabilitation, GRAIL

## Abstract

**Background:**

Patients with cerebellar malformations exhibit not only movement problems, but also important deficits in social cognition. Thus, rehabilitation approaches should not only involve the recovery of motor function but also of higher-order abilities such as processing of social stimuli. In keeping with the general role of the cerebellum in anticipating and predicting events, we used a VR-based rehabilitation system to implement a social cognition intensive training specifically tailored to improve predictive abilities in social scenarios (VR-Spirit).

**Methods/design:**

The study is an interventional randomised controlled trial that aims to recruit 42 children, adolescents and young adults with congenital cerebellar malformations, randomly allocated to the experimental group or the active control group. The experimental group is administered the VR-Spirit, requiring the participants to compete with different avatars in the reaching of recreational equipment and implicitly prompting them to form expectations about their playing preference. The active control group participates in a VR-training with standard games currently adopted for motor rehabilitation. Both trainings are composed by eight 45-min sessions and are administered in the GRAIL VR laboratory (Motekforce Link, Netherlands), an integrated platform that allows patients to move in natural and attractive VR environments. An evaluation session in VR with the same paradigm used in the VR-Spirit but implemented in a different scenario is administered at the beginning (T0) of the two trainings (T1) and at the end (T2). Moreover, a battery of neurocognitive tests spanning different domains is administered to all participants at T0, T2 and in a follow-up session after 2 months from the end of the two trainings (T3).

**Discussion:**

This study offers a novel approach for rehabilitation based on specific neural mechanisms of the cerebellum. We aim to investigate the feasibility and efficacy of a new, intensive, social cognition training in a sample of Italian patients aged 7–25 years with congenital cerebellar malformations. We expect that VR-Spirit could enhance social prediction ability and indirectly improve cognitive performance in diverse domains. Moreover, through the comparison with a VR-active control training we aim to verify the specificity of VR-Spirit in improving social perception skills.

**Trial registration:**

ISRCTN, ID: ISRCTN 22332873. Retrospectively registered on 12 March 2018.

## Background

Previous research has explored the involvement of the cerebellum in a wide range of motor and cognitive functions. In particular, there is a general agreement that the cerebellum plays a crucial role in detecting contextual regularities and sequence and in forming and updating internal models of external events [1–3]. Cerebellar neuroanatomical and topographical organisations, with a uniform neuronal structure but multiple functional connections with other brain areas, are in line with its role as an effective forward controller through internal models [4–6]. Indeed, it has been demonstrated that different populations of cerebellar cells encode for sensorimotor and non-sensorimotor predictions and for their violations [7–10]. Accordingly, this predictive computational mechanism has been generalised to multiple domains, from a simple eye-blinking response to complex social behaviours [2, 3, 11]. Importantly, the cerebellar contributes to different domains have been proposed as a unified theoretical framework that could shed new light on the complex pattern of motor, cognitive, linguistic and behavioural disorders identified as cerebellar cognitive affective syndrome (CCAS [3, 6, 12];).

Given the increasing weight attributed to prediction mechanisms in neurocognitive models of how we understand others’ intentions [13], recent studies have focussed on the role of the cerebellum in social cognition [14, 15], namely the set of mental processes that are needed to understand social interactions and regulate social behaviour [16]. Research has confirmed that cerebellar diseases are associated with alterations in crucial aspects of social cognition, such as theory of mind [17, 18] and emotional processing [19]. Through its connection with cortical areas involved in the mentalising networks [20], the cerebellum seems to play a critical role in sequencing other people’s behaviours, resulting in the formation of predictive internal models of social events that are then matched with external information [21, 22]. Thus, alterations of the cerebro-cerebellar loops in congenital cerebellar malformations may affect predictive mechanisms, resulting in the impaired ability to understand others’ intentions, social deficits, and autism-like behaviours [23, 24].

In this light, rehabilitation programmes for children and adolescents affected by congenital cerebellar malformation should not only involve the recovery of motor functions, but also of higher-order abilities, such as cognitive processing of social stimuli [25]. However, only few single-case studies have reported data concerning cognitive rehabilitation for patients with cerebellar diseases, either congenital [26] or acquired [27–30]. Crucially, none of these previous studies has focussed on social cognition and has exploited the boosting of the specific predictive abilities of the cerebellum in order to treat and rehabilitate the neuropsychiatric symptoms shown by patients with cerebellar dysfunction [31].

Virtual reality (VR) has been proposed as a useful tool for the assessment, treatment and rehabilitation of mental health disorders due to the embodied experience and natural sense of presence offered by this technology [32–34]). Recently, innovative interventions using VR have been proposed for social skills’ training in adult and paediatric patients with autism spectrum disorders (ASDs) [35, 36] and with schizophrenia [37]. Immersive VR appears to be a promising field for cognitive and socio-emotional rehabilitation, especially for the paediatric age group, because of its highly motivating and interactive nature [38, 39].

Here, we use the GRAIL VR laboratory (Motekforce Link, Amsterdam, Netherlands) to develop a brand-new social cognition intensive training (VR-Spirit) based on the specific computation exerted by the cerebellum in predicting others’ behaviour. The GRAIL technology has been developed for motor rehabilitation, specifically for gait analysis and training, and is equipped with a dual-belt treadmill, a motion platform and an integrated motion-capture system. VR environments are projected on to a 180° cylindrical screen running in synchronisation with the treadmill in order to create a natural optic flow. This system has been already adopted for the motor assessment and rehabilitation of paediatric patients [40, 41]. Furthermore, recent studies have indicated that immersive VR technology could be useful even in cognitive rehabilitation [42] and behavioural interventions [43, 44].

For the aim of the present project, we developed two different VR scenarios, namely ‘playground’ and ‘sweet stands’, respectively, for the training and for the evaluation sessions. Participants are engaged in a competition with one of four avatars, who are easily identifiable by their clothing and body cues, and they have to reach one of three pieces of recreational equipment (training) or one of three stands (evaluation) before the avatar. On each trial, the participants are given a score when they arrive before the avatar to the game/stand mostly chosen by themselves during the course of the session. Social scenarios have been designed to force the children to anticipate avatars’ movements, thus predicting their preference. Four avatars were created for each scenario and were associated with pre-established probabilities to prefer each game/stand, thus showing a clear preference for one of the objects. Four different sessions were generated in order to equally distribute probability associations between avatars and objects and were presented in the 4 days of each training week. Hence, the avatars have different probabilities in each session with the purpose of avoiding possible memory effects. Day by day, experimental group patients are expected to implicitly learn the associations between contextual cues (games/stand) and avatars’ intentions in that session and anticipate their choice. In other words, the paradigm that underlies our applications should specifically enhance the building of predictive internal models of others’ behaviour. Since a lack of this computation has been proposed to explain social deficits in patients with cerebellar dysfunction [15, 31] as well as in autism [45], boosting the building of internal models of others’ intention could improve social prediction with a likely impact on more general social cognition abilities.

## Methods/design

### Aim

The primary aim of the trial is to investigate the efficacy of a new, intensive, cognitive rehabilitation protocol in a sample of Italian patients aged 7–25 years with congenital cerebellar diseases. The hypothesis is that the VR-Spirit rehabilitation protocol should:

1. Enhance social prediction ability resulting in better understanding of other people’s intentions and behaviours
2. Facilitate general-domain implicit-learning ability
3. Indirectly improve cognitive performance in specific domains (attention and executive functions, memory, visuospatial abilities, sensorimotor integration)
4. Produce an amelioration of patients’ quality of life

Contextually to the primary aim, the study would verify the feasibility and acceptability of this rehabilitative intervention for the target population.

### Participants

Participants are children, adolescents and young adults aged 7–25 years with congenital cerebellar malformations and with a Full-scale Intelligent Quotient (FSIQ) greater than 45. Cerebellar malformations refer to anatomical abnormalities affecting the vermis and/or the hemispheres not due to acquired lesions and not associated with progressive pathologies. However, it is noteworthy that these patients could exhibit malformations in other cortical structures. As an example, patients with Joubert syndrome often present with malformations of the pontine and medullary areas [46]. Participants are recruited at the Child Neuropsychiatry and Neurorehabilitation Unit of the Scientific Institute, IRCCS E. Medea. With the aim of achieving the target sample size, associations of patients can be contacted. The following exclusion criteria have been adopted:

1. Severe sensorial, motor and/or behavioural problems that could interfere with the use of GRAIL technology
2. Being simultaneously involved in a different cognitive rehabilitation treatment, to avoid excessive demands to children and possible interference on training adherence rates
3. Having been involved in a different cognitive rehabilitation treatment in the last 6 months before training, to avoid confounding follow-up effects

Parents of all known potentially eligible patients are contacted telephonically by the attending physician and are informed about the aims and methods of the protocol. If they agree with the study, an administrative staff member contacts the parents to organise the recovery. Since participation in the trial has to be arranged according to other clinical needs (e.g. routine medical checks) of the patients and to parents’ availability, a variable time frame from 1 month to 1 year could occur before patients get access to the trial. Intervention assignment is carried out by a research assistant only when patients are admitted to the recovery. It is noteworthy that the administrative staff that attends to the recruitment and organises hospitalisation is blinded to group allocation. Before starting any study procedures, a research assistant provides a description of the material and procedures to parents and patients and asks them to sign an informed consent.

### Design

The study applies a single-centre, randomised, active-controlled trial design. Patients are allocated to one of two groups undergoing two different rehabilitation programmes through a stratified permuted block randomisation procedure [47]. Age and cognitive level (more recently available Full-scale Intelligent Quotient, FSIQ) are chosen as stratification factors. In particular, we consider two levels for age, corresponding to 7.0–12.9 and ≥ 13.0 years, and three stages for cognitive level consistent with, respectively, absence of intellectual disability (FSIQ > 80), from borderline intellectual functioning to mild intellectual disability (80 ≥ FSIQ ≥ 61) and from mild to moderate intellectual disability (60 ≥ FSIQ ≥ 45) (48). Doing so, six blocks are generated and within each block an estimated number of eight patients should be enrolled to achieve and overcome the established sample size. First, participants are allocated to a block depending on the two stratification variables. Second, patients are assigned to one of the two interventions according to specific permuted sequence. Details of the permuted blocks are reported in Table 1.

**Table 1:** Stratified permuted blocks randomisation of the study

		Age	
		7–12.9 years	≥ 13.0 years
FSIQ	46–60	CCSSCCSS	SCSCSCSC
	61–80	SCCSSCCS	SSCCSSCC
	> 80	CSCSCSCS	CSSCCSSC

Legend: *FSIQ* Full-Scale Intelligent Quotient, *S* VR-Spirit training, *C* control training

Group 1 (S) receives the social prediction VR training for 2 weeks (four daily sessions in a week). In each 1-h session, 80 trials of the experimental programme and one of four motor games, selected among the applications available in the GRAIL, are administered; for each weekly session, a different game is administered in random order. Group 2 (C) receives a control VR training of the same duration (2 weeks, four 1-h sessions per week) as the experimental training; the control training involves, for each session, a navigational game and the daily repetition of all the four games from the GRAIL suite that are also presented, one per day, in the experimental session; the social prediction experimental programme is not presented in the control training.

Before the training (T0), a battery of neurocognitive tests from the Developmental NEuroPSYchological Assessment, 2nd edition (NEPSY-II [49, 50];) spanning different domains, and, specifically, social perception abilities, is administered to all participants. Both groups also receive a 10-min training of how to move within the GRAIL environment using a custom-made navigational application. Then, a pre-training evaluation through a VR game session, based on the same paradigm of the VR experimental training but in a different scenario, and a computer-based Action Prediction task [51] are administered. Moreover, at T0, both patients and parents compile questionnaires on quality of life (Tno-Azl (Netherlands Organisation for Applied Scientific Research Academic Medical Centre) Children’s Quality Of Life questionnaire (TACQOL), TNO Quality of life/LUMC, 2001 [52];) and parents also complete the Child Behaviour Check List (CBCL [53, 54];).

Performance at the VR tasks is monitored for all the sessions of the training period (T1). In order to verify and compare the effects of the experimental and control training sessions, at the end of the 2-week training (T2) all participants are re-evaluated with the same neurocognitive tests, the VR evaluation scenario and the Action Prediction task.

**Fig. 1:**
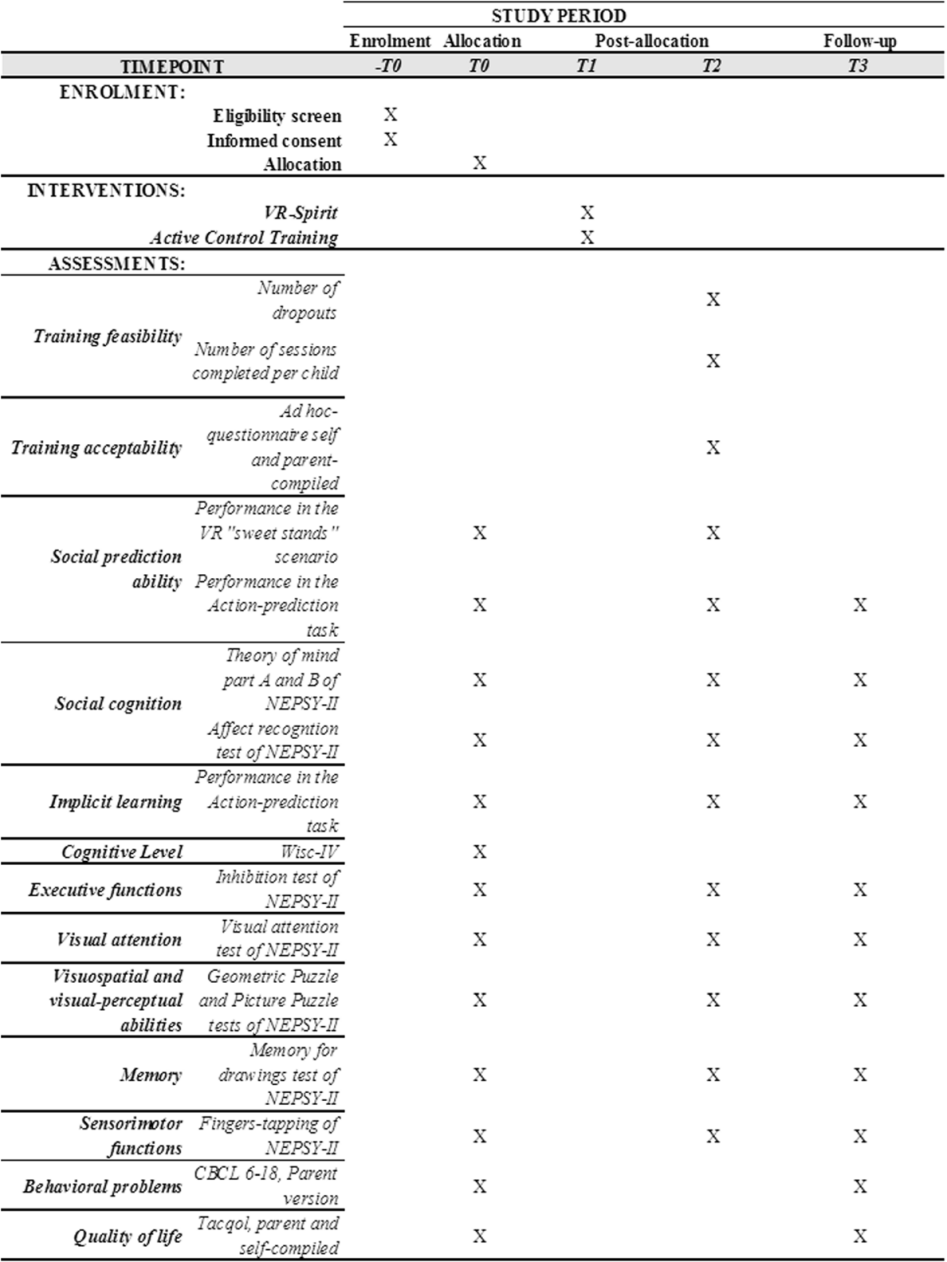
Study design

Legend: *VR* virtual reality, *NEPSY-II* Developmental NEuroPSYchological Assessment, 2nd edition, *WISC-IV* Wechsler Intelligence Scale for Children, 4th edition, *CBCL* Child Behavior Check List, *TACQOL* (TA) Children’s Quality Of Life questionnaire

With the aim to investigate the far transferability of the effects, a follow-up evaluation is provided after 2 months (T3) with the same protocol used at T0 except for the GRAIL evaluation scenario. Details of the study design are set out in Fig. 1 according to the Standard Protocol Items: Recommendations for Interventional Trials (SPIRIT) Statement [55, 56] (see also Additional file 1).

### Intervention and study setting

The rehabilitation trainings are administered in the GRAIL laboratory at the Scientific Institute (IRCCS) E. Medea (Bosisio Parini, Italy). The GRAIL system is an integrated platform equipped with a treadmill on a motion frame, a Vicon motion-capture system (Oxford Metrics, Oxford, UK) and a 180° cylindrical projection screen. The D-flow software controls the relationship between the patient, the scenery and the interactive feedbacks and stimulations. This software runs on Microsoft Windows and it was used to develop the interactive VR applications with a block diagram approach. For the creation of the GRAIL scenes, objects and scenario were modelled separately by means of Google SketchUp while the avatars were created by using MakeHuman and then modified in Blender. The modelling process was first dedicated to the creation of three-dimensional geometries and then to the application of selected materials and textures. Files generated in SketchUp and Blender were exported in the COLLADA interchange format and then imported into Autodesk 3ds Max software. The latter allowed us to convert models in Wavefront OBJ format and to assemble all the models created within the scenery. The whole scene was exported in Ogre format to be used within the D-flow software: the final scene contained the environment and the individual objects.

**Fig. 2:**
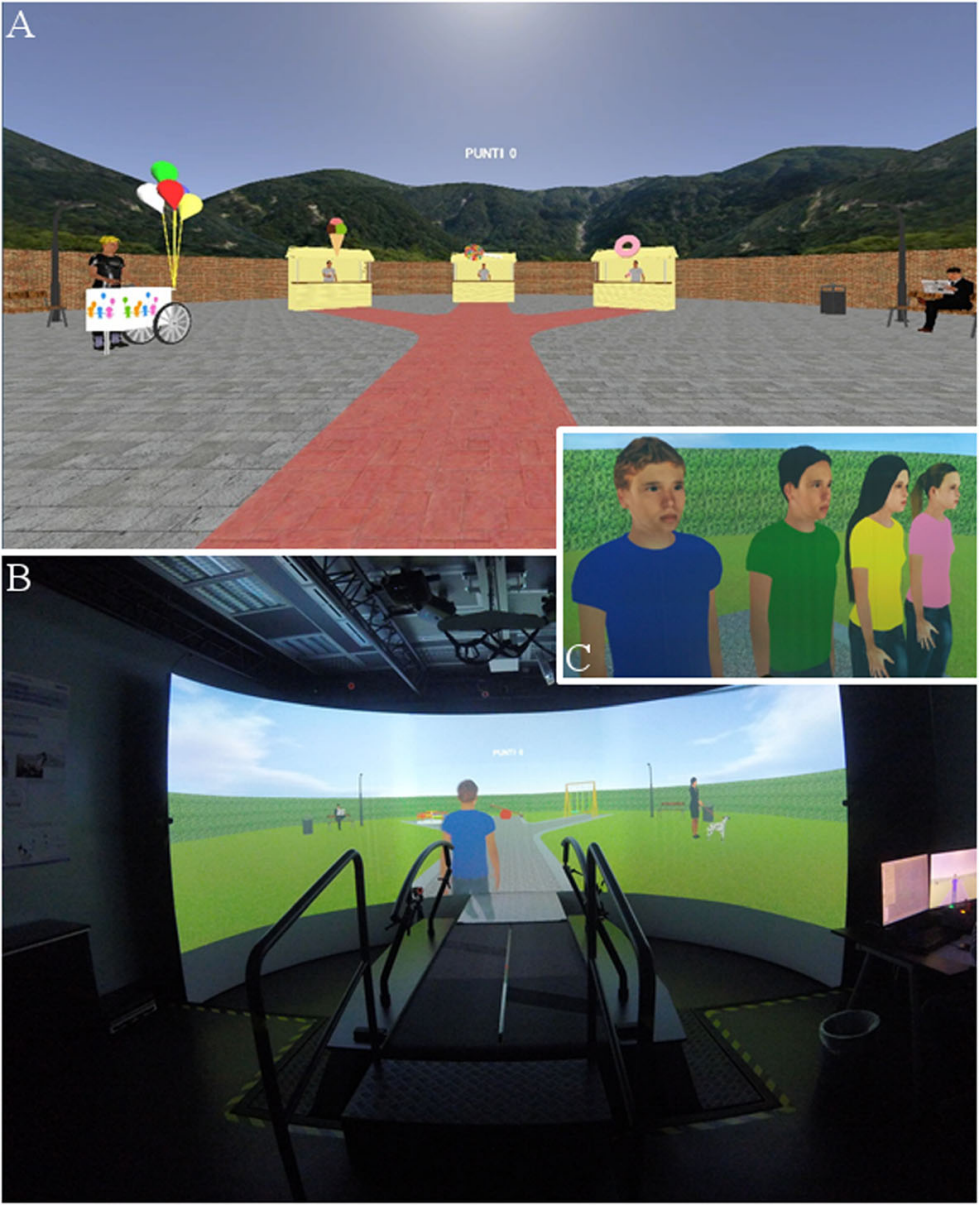
**a** The virtual reality (VR) environment designed for the evaluation sessions. **b** The GRAIL platform and the VR application running a training session; one of the avatars is visible to the subject. **c** The four avatars used during the training session

Two different scenes were developed specifically for this study: the sweet stands’ environment for the pre- and post-training evaluations and the playground scenario for the social prediction training. Both scenes are designed with a linear, 9-m-long path that branches into three 3-m-long streets. At the end of each branch, one of three objects is located in a semicircle at the same distance from the starting point: the playground setting includes a swing, a circular carousel and a rocking carousel, while the sweet stands’ setting includes an ice cream, a donut and a lollipop stand. Furthermore, four different avatars, two male and two female, were designed: they are adolescents, clearly identifiable by body and clothing features (i.e. hair and t-shirt colours). An example of the two scenarios and of the avatars, respectively, for the evaluation and for the training sessions, is reported in Fig. 2.

Before the beginning of the session, the patient wears two reflective markers on the posterior superior iliac spines, that allow the tracing of the patient’s movements and control the virtual environment: to go faster, the patient has to move forward, to slow down they have to move backward, while to turn right or left they have to shift the pelvis right or left. Then, the patient comes up the GRAIL system, the trainer calibrates their starting position and the session can start. Before starting the first evaluation session, a short and effective navigational training is administered to participants with the aim of learning to navigate within the GRAIL VR environments. A physical therapist specifically patented for using GRAIL technology administers all the applications.

### Evaluation sessions

Pre- and post-training evaluation sessions in the sweet stand ’scenario are administered to both the experimental and the control groups. The paradigm exploits a probabilistic design that has already shown its reliability in assessing social prediction abilities, since children with ASD, who show clinically relevant social deficits, were impaired in using contextual priors to predict the unfolding intention of observed actions ahead of realisation [45]. Within a session, events take place in a pseudorandom way in respect to the pre-established probabilities. Specifically, in each trial, one of four avatar moves from the starting point to one of the stands with pre-established probabilities as shown in Table 2:

**Table 2:** Example of event probability in a evaluation session with the “sweet stands” scenario

Session A	Stand		
Avatar	Ice cream	Donut	Lollipop
Avatar A	80%	10%	10%
Avatar B	10%	80%	10%
Avatar C	10%	10%	80%
Avatar D	33%	33%	33%

Two different evaluation sessions were generated, changing the avatar-object associations and event sequence, and are presented, respectively, at T0 and T2 in random order. In this way, we avoid repetition of the same events in the two evaluation sessions in order to minimise learning effects. The order of the two sessions is counterbalanced between patients of the same group (e.g. for patient 1 session A at T0 and session B at T2, for patient 2 session B at T0 and session A at T2, etc.). Considering 20 trials per avatar, 80 trials are administered in each session.

The GRAIL therapist asks the patient to move toward the sweet stand chosen by the avatar and activate it before them. In each trial the patient has to reach one of the stands in maximum 15 s and their maximum speed is 2 m/s. The avatar, one per trial and visibly positioned next to the patient, moves towards a stand, reaching it in 10 s. When the participant reaches a sweet stand, the stand is activated providing a visual reinforcement, otherwise the event is interrupted 5 s after the avatar has reached the object and the patients are invited to try again. Furthermore, when the participant anticipates the avatar in reaching the correct stand they also receive an auditory reinforcement (clapping sound), which signals the scoring of a point in the game in addition to visual reinforcement (activation of the object). The object reached by the avatar is always visible to the participant, for both successful and unsuccessful trials, in order to provide information on the avatar’s preferences that can be used in the next trial. Specific features of the application force the patients to move according to the anticipation of the avatars’ preference rather than following their movements. Indeed, the path is a 9-m straight-line trajectory and, then, it splits into three ways. Therefore, participants are not exposed to motion cues concerning avatars’ directions until the crossroad. Moreover, after this division the speed of the avatars equals the maximum available for the patients, so that they cannot be surpassed anymore. This way, patients are prompted to implicitly learn the probabilistic associations between the avatar and the most chosen sweet stand, thus allowing this paradigm to evaluate and improve the ability to form predictive models of others’ behaviour.

For each trial, the D-flow software automatically saves one raw with the following measures in a .txt file:

- Duration of the trial
- Mean speed of the subject
- Speed of the subject at specific points of the path (e.g. at 0.5, 1, and 9 m from the starting point)
- Specific avatar
- Object selected by the avatar
- Object selected by the subject
- Victory/no victory
- Incremental score

At the end of the 80 trials, the D-flow automatically saves the total score of the session in a different .txt file.

### VR-Spirit training

Training sessions adopt the same logic as the evaluation sessions but in the playground scenario. With the aim to balance the association between avatars and objects, four diverse sessions (A, B, C, D) were obtained, such that avatars’ probability of moving toward a specific object is equally distributed across the four sessions. The four sessions are randomly administered during the first week and repeated in the same order in the second week.

Every day, the experimental group is administered with one of the four diverse sessions of the VR-Spirit training so that avatars’ preferences change day by day both in the first and in the second week. Moreover, after the participants have completed the 80 social prediction trials, they also play one of four selected games from the GRAIL kit (see below for the description of the games).

### Active control training

The control group is exposed for the same amount of time (1-h session per day, four sessions per week for 2 weeks) to sessions requiring the participants to play a navigational game, in which they have to conduct a ball out of three mazes, and all four selected GRAIL games. The four selected games are ‘skiing’, ‘balloon shooting’, ‘world soccer’ and ‘traffic jam’. These games have been chosen because they do not present social agents and do not require any form of prediction ability. In the skiing game, participants have to do a slalom between snowmen, scoring a point when they pass each snowman on the right side. In the balloon shooting game, participants have to hit balloons appearing in a natural environment simply by pointing at them. In the world soccer game, children kick a virtual ball toward a goal: they score points when they hit targets put inside the goal. In the traffic jam game, participants are in the middle of a crossroad and they have to raise their left or right foot according to the cars’ movements.

### Outcome measures

Primary outcome measure:

1. Social prediction ability: performance during the pre- (T0) and post-training (T2) evaluation in the sweet stands’ scenario; standardised beta-coefficients of the regression between accuracy and probability in the testing phase of the validated PC-based Action Prediction task administered before/after the intervention (T0, T2) and at the follow-up visit (T3)

Secondary outcome measures:

1. Social cognition: Theory of Mind Parts A and B and Emotion Recognition of the NEPSY-II testing battery
2. Implicit learning: accuracy and reaction time in the familiarisation phase of an Action Prediction task
3. Executive functions (inhibition and flexibility): Inhibition test of NEPSY-II
4. Visual attention: Visual Attention test of NEPSY-II
5. Visuospatial and visual-perceptual abilities: Geometric Puzzle and Picture Puzzle tests of NEPSY-II
6. Memory: Memory for Drawings test of NEPSY-II
7. Sensorimotor functions: Finger-tapping of NEPSY-II
8. Behavioural problems: CBCL 6–18, Parent version
9. Overall functioning and quality of life assessed using the TACQOL questionnaire, presented in two forms: the self-compiled one and the parent-compiled one

To assess training feasibility:

1. Number of dropouts: number of children who renounce to complete the 2-week training
2. Number of sessions completed per child: total number of sessions performed in front of the total number proposed of eight sessions

To assess training acceptability:

1. Acceptability questionnaire: an ad-hoc questionnaire completed by participants and another one by their parents after training conclusion (T2) to assess subjective evaluation of training accessibility and efficacy. It is to note that the same questionnaires are fulfilled by the patients of the active control group and their parents

A psychologist, who is not blinded to the intervention assignment, administers all the neuropsychological tests and questionnaires and records the performance during the GRAIL sessions.

### Action Prediction task

We adopt a validated, computer-based Action Prediction task as an experimental outcome measure for social prediction and implicit-learning abilities. This experimental paradigm consists of a probabilistic learning task (familiarisation phase) followed by an Action Prediction task (testing phase). During familiarisation, participants are exposed to videos showing a child actor performing two different grasping actions associated with specific contextual cues and they are asked to recognise the actor’s intention. Notably, in this phase the association between contextual cues and actions was implicitly biased with pre-established probability of co-occurrence. During testing, the second half of the same videos is occluded and the patients are asked to predict the final outcome of the action. Since movement kinematics are ambiguous, responses should be biased toward the contextual priors acquired during the familiarisation phase. For each participant and separately for the two phases, a standardised beta-coefficient is calculated across the trials at the individual level using a regression analysis with probability and accuracy as the independent and dependent variables, respectively. Indeed, the beta-coefficient could be considered as a direct index of the strength of the contextual models of others’ intentions, thus providing a measure of social prediction ability.

### Neuropsychological assessment

A neuropsychological assessment is administered at each stage of the study using the Italian version of the NEPSY-II battery. The NEPSY-II is designed to evaluate six different cognitive domains in children and adolescents aged 3–16 years. In our study, we administer tests that assess visual attention and executive functions, visuospatial memory and functions, sensorimotor integration and social perception skills. The Visual Attention test assesses speed and accuracy of patients in focussing and maintaining attention on visual targets among a series of distracting stimuli. To assess executive functions, we adopt the Inhibition test, in which participants are asked to denominate different figures respecting diverse rules, thus inhibiting automatic responses. In the Memory for Drawing test, children are exposed for 10 s to a table representing drawings in diverse spatial positions and then they are asked to choose the correct stimuli in a series of cards and place them in a panel in the same position that they have seen before. The recall is asked immediately after the exposition and after 20 min. The Picture and the Geometric puzzles use, respectively, concrete and abstract examples to evaluate visual-perceptual and visual-spatial representation abilities. For sensorimotor integration, we administer the Finger-tapping test, which measures the ability to repeat fast finger movements and maintain a motor programme. To assess social perception skills, we administer the Theory of Mind and the Affect Recognition tests. The first is composed of two parts resulting in one score. In the verbal part, verbal or pictorial descriptions of social situations are presented in order to evaluate the ability to understand mental constructs, such as beliefs and intentions, and how other people could have thoughts, emotions and perspectives, which might be different from ours. Conversely, the contextual part assesses the ability to infer others’ emotional and mental states by social context. The Affect Recognition test provides a measure of the ability to recognise affective states from emotional facial expressions using pictures of children. Moreover, the baseline assessment includes a full cognitive evaluation with the Wechsler Intelligence Scale for Children, 4th edition (WISC-IV) to estimate IQ scores [57].

### Questionnaires

At the baseline (T0) and follow-up evaluations (T3) the TACQOL is administered to parents and children. This questionnaire has been primarily designed for research and evaluates quality of life in diverse domains: body, movements, autonomy, cognitive abilities, sociality and positive and negative emotionality. At the same time points, parents are asked to compile the CBCL, the most adopted questionnaire regarding the behaviour of children and adolescents. This questionnaire provides scores for eight empirically based syndrome scales, namely aggressive behaviour, anxiety/depressive symptoms, attention problems, rule-breaking behaviour, somatic complaints, social problems, thought problems and degree of withdrawal. These scales are further aggregated in three main dimensions: internalising, externalising and total problems.

### Statistical methods

Demographic, clinical and neuropsychological variables of the two groups of patients are inspected through descriptive statistics. A *t* test and *χ*^2^ are used to assess the differences between the experimental and control training groups at baseline for continuous and categorical variables, respectively, thus allowing us to verify successful randomisation. For each outcome measure, we calculate the change between T0 and T2 (training effect delta) and between T0 and T3 (follow-up effect). Delta is calculated as the arithmetic difference between the second/third time points and the first time point. The delta values of the two groups for the primary outcome measure are compared using independent sample *t* tests (two-tailed). For the primary outcome measure, a Bonferroni correction procedure will be used to control for multiple comparisons. Multivariate analysis of variance will be used to explore differential effects of the trainings in the secondary outcome measures. As to what concerns missing data, a modified intention-to-treat analysis approach will be adopted, including in the analyses all the participants who had completed the pre- and post-treatment evaluation sessions, even if they had not completed all the training sessions. No imputation of missing data, however, will be used considering the limited sample size and observation points [58, 59]. The stratification factors (i.e. age and FSIQ), will not be considered in the statistical analyses, assuming that the stratification procedure has ensured a balanced distribution across the two groups.

### Estimation of sample size

Estimation of sample size was based on the distribution of the standardized beta-coefficients of the regression between accuracy and probability in the PC-based, contextualised Action Prediction task in children with typical development (M = 0.154, SD = 0.215) and with ASD (M = 0.008, SD = 0.112), with a between-group difference of moderate-to-large size (Cohen’s *d* = 0.87; see Amoruso et al., 2019 [45]). Given the difference in the standardised beta-coefficient between children with typical development and with ASD, an increase of moderate effect size in this outcome measure should represent a clinically relevant improvement in the ability to form predictive models of others’ behaviour, with a likely impact on social deficits and autism-like behaviours shown by patients with cerebellar malformations. Thus, we estimated that a moderate increase (0.13 mean change or 0.8 SD) of the standardised beta-coefficient after as compared to before the experimental training had clinical significance. Accordingly, a final sample of 21 patients per group has been set for our study in order to detect a between-group difference (independent sample *t t*est, two-tailed) between the effects of the experimental vs*.* control training (T2 − T0) of moderate effect size (Cohen’s *d* = 0.8) with a power of 0.80 and an alpha level set at 0.05. The software G Power 3 was used for this estimation.

## Discussion

Evidence on the role of the cerebellum in social cognition is rapidly increasing, showing that a dysfunction of core predictive mechanisms could impact on high-level social-cognitive abilities, such as Theory of Mind and the processing of social stimuli [15, 19]. These findings are in line with clinical reports on the importance of behavioural, affective and social skill alterations presented by patients with cerebellar diseases [12, 31, 60, 61]. Though, available rehabilitative interventions for these pathologies typically address motor and cognitive alterations [26, 28–30], but not social cognition deficits. Our study tries to fill this gap, proposing a protocol that aims to improve social prediction skills.

Rehabilitation treatments based on specific neural mechanisms of the brain offer the opportunity to design interventions with a clear rationale, but they also directly serve as a clinical validation of theoretical knowledge on brain functioning [62]. Other researchers have developed a brain-based VR treatment for adult patients [63], while VR-Spirit is a rehabilitative protocol targeted at children, adolescents and young adults specifically designed on the core predictive mechanism of the cerebellum. Notably, this kind of interventions appears particularly important in this developmental age group as it could benefit from natural brain plasticity [64, 65], fostering an effective impact on the quality of life and social participation of these patients.

The study design provides for an active control group that participates in a training in VR, playing games already adopted for motor rehabilitation. On one hand, this approach allows us to verify the specificity of the social prediction training in improving social cognition abilities. On the other, it enables us to investigate the effects of VR interventions on other cognitive abilities indirectly involved in the two trainings. Indeed, intrinsic features of VR systems, such as the natural sense of presence, the movement in attractive scenarios and the delivery of complex, multisensorial feedbacks, could enhance visual-spatial ability and sensorimotor integration [32]. However, since both the experimental and the control trainings engage motor systems, any differential outcome of the VR-Spirit vs. the control training is unlikely to reflect motor symptom improvements. Nevertheless, any change in sensorimotor functions as assessed by NEPSY-II subtests should be considered and controlled when analysing and discussing the outcomes of the study.

The VR-Spirit protocol provides a new kind of interventions for neurorehabilitation of patients with cerebellar malformations. While it fills a gap of rehabilitation on social cognition, it is worth mentioning that this approach could only partially address the rehabilitative needs of children, adolescents and young adults with congenital cerebellar diseases, which should necessarily encompass other interventions focussed on the motor [66] and cognitive deficits typically shown by these patients [61]. However, VR-Spirit is a first step to design future rehabilitative protocols targeted on the different clinical manifestations of CCAS.

The literature has widely documented cerebellar engagement in social cognition tasks in both low-level and high-level processing [15, 67]. Accordingly, we could reasonably assume that VR-Spirit activates the cerebellum along with other cortical areas involved by social inference [20]. Although, we did not directly verify this hypothesis. Future studies should consider adopting neurophysiological measures (e.g*.* electroencephalography) in order to investigate whether and how the cerebellum and other cortical areas are engaged by our paradigm.

Previous research has suggested that an intensive, goal-directed treatment could be at least as effective as one administered over a longer time and it could also be more acceptable by patients and their parents [26]. Although our protocol is in line with the state of the art in this field, the short duration and the required hospitalisation may limit the possibility of controlling the efficacy of this intervention on everyday life. Further, the use of expensive technology makes it difficult to replicate and extend the training to a wider sample. Future research may implement the VR-Spirit training using more accessible and wearable devices (e.g. X-Box Kinect and VR head-mounted displays), that allow it to be administered it in more ecological contexts.

The GRAIL technology is a medical device with 93/42EEC certification provided with safety equipment to prevent any risk associated with its use. However, VR treatments could be accompanied by unwanted side effects such as nausea and headache, which are referred to as simulator sickness or cyber sickness [68]. Although previous studies adopting GRAIL demonstrated its acceptability by clinical paediatric samples [40, 69], in case a participant should present cyber sickness symptoms the session will be interrupted until their sickness ends. Any episode of cyber sickness will be recorded by the GRAIL therapist and considered for evaluating the feasibility and acceptability of the intervention.

Finally, it is noteworthy that the participants are randomly allocated to the two groups, but the psychologist administering the neuropsychological evaluation, the GRAIL therapist and the parents of the patients are not blinded to the typology of interventions. As what concerns randomisation, the use of stratified randomisation has been issued for introducing biases when baseline features of all participants are not available before assignment [70]; nevertheless, it allows control of the effects of influencing covariates in a small sample size [71]. Moreover, while we reduced as much as possible the strata based on the importance of the clinical variables, the availability of patients for our sample may not consent to fulfilling all the blocks [47]. These methodological issues should be considered and discussed when evaluating and interpreting the results of the protocol. The results on efficacy and feasibility will be published in international peer-reviewed journals. Preliminary data on efficacy and feasibility will be submitted when at least half of the target sample has been recruited. Final efficacy results will be submitted within 6 months after the end of the trial.

## Trial status

This protocol has been applied to ISRCTN registry on 12 January 2018, registered on 12 March 2018 and last edited on 20 December 2018. Recruitment started in February 2018 and will be continuing until August 2020 and is currently recruiting.

## Supplementary information

**Additional file 1.** Standard Protocol Items: Recommendations for Interventional Trials (SPIRIT) Checklist.

## Data Availability

Data are collected in a protected database and are anonymised, as a research member assigns to each participant an identity number that substitutes the name. Participants’ parents give written informed consent to anonymised data use. The anonymised data are available on request by sending an email to Dr. Renato Borgatti (renato.borgatti@lanostrafamiglia.it). All data will be available for 5 years after the relevant publication.

## Abbreviations

CBCL: Child Behaviour Check List
CCAS: Cerebellar cognitive affective syndrome
FSIQ: Full-scale Intelligent Quotient
NEPSY-II: Developmental NEuroPSYchological Assessment, 2nd edition
TACQOL: Tno-Azl (Netherlands Organisation for Applied Scientific Research Academic Medical Centre) Children’s Quality Of Life questionnaire
VR: Virtual reality
WISC-IV: Wechsler Intelligence Scale for children, 4th edition
